# Associations Between Metabolic Profiles and Target-Organ Damage in Chinese Individuals With Primary Aldosteronism

**DOI:** 10.3389/fendo.2020.547356

**Published:** 2020-09-25

**Authors:** Shao-Ling Zhang, Jing-Wei Gao, Ying Guo, Qi-Ling Feng, Ju-Ying Tang, Li Yan, Jing-Feng Wang, Hua Cheng, Pin-Ming Liu

**Affiliations:** ^1^Department of Endocrinology, Sun Yat-sen Memorial Hospital of Sun Yat-sen University, Guangzhou, China; ^2^Department of Cardiology, Sun Yat-sen Memorial Hospital of Sun Yat-sen University, Guangzhou, China

**Keywords:** primary aldosteronism, metabolic syndrome, association, target-organ damage, essential hypertension

## Abstract

**Purpose:** Patients with primary aldosteronism (PA) have an increased risk of target-organ damage (TOD), but whether metabolic syndrome (MetS) is more prevalent and contributes to TOD in PA patients remains unresolved. We aimed to evaluate the associations between MetS profiles and TOD in Chinese PA individuals.

**Methods:** Metabolic parameters and pre-clinical TOD including left ventricular hypertrophy, estimated glomerular filtration, and microalbuminuria; insulin sensitivity or resistance; and islet β-cell function were assessed by the homeostasis models (HOMA-IR, HOMA-β) and the other surrogate indexes [composite insulin sensitivity index (ISI), modified β-cell function index (MBCI)] determined from the oral glucose tolerance test were compared in PA vs. matched essential hypertension (EH) patients.

**Results:** A total of 109 PA patients and 109 essential hypertension (EH) controls individually matched for sex, age, and office systolic blood pressure and duration of hypertension were studied. The prevalence of MetS and its individual components in PA was significantly lower than in EH [MetS: 28 (25.6%) vs. 54 (49.5%), *P* < 0.001]. PA patients had a higher composite ISI but a lower HOMA-IR, HOMA-β, and MBCI than EH controls (all *P* < 0.05). Concerning TOD, PA patients had significantly higher prevalence of microalbuminuria and left ventricular hypertrophy (LVH), and lower levels of estimated glomerular filtration (eGFR) than EH controls (all *P* < 0.05). On multivariate logistic regression analysis, female gender and elevated plasma aldosterone levels were significantly associated with TOD in PA. However, there were no significant associations between MetS and its individual components and TOD in PA patients.

**Conclusions:** PA patients had a lower MetS prevalence but exhibited more severe TOD than matched EH controls. The study highlights the deleterious effects of aldosterone excess on the development of TOD, whereas MetS or its individual components might be less influential in PA.

## Introduction

Primary aldosteronism (PA) is the most frequent cause of endocrine hypertension, characterized by the autonomous aldosterone overproduction followed by suppressed plasma renin activity (PRA). Investigations of different populations suggest that PA affects about 10% of unselected patients with hypertension (1) and is particularly common in patients with resistant hypertension, with prevalence as high as 20% (2). Considering the fact that approximately 245 million adult people in China are currently hypertensive (3), it can be roughly estimated that at least 20 million people may be affected by PA. The two predominant PA subtypes are aldosterone-producing adenoma (APA) and bilateral idiopathic hyperaldosteronism (IHA) (1). Compared with age-, sex-, and blood pressure (BP)-matched patients with essential hypertension (EH), PA patients have an increased risk of cardiovascular events, including stroke, myocardial infarction, diastolic dysfunction and heart failure, and atrial fibrillation (4, 5), which is closely associated with antecedent pre-clinical target-organ damage (TOD) and increased cardiovascular remodeling, such as left ventricular hypertrophy (LVH), artery stiffness and calcification, widespread tissue inflammation, and fibrosis (6, 7).

Metabolic syndrome (MetS), manifested as abdominal obesity, raised triglycerides (TG), reduced high-density lipoprotein cholesterol (HDL-C), and hyperglycemia is a common feature in hypertensive patients. A cluster of these cardiovascular risk factors will further increase cardiovascular morbidity and mortality (8). Studies on comparison of MetS prevalence between PA and EH patients have generated discordant results during the past decades (9–12). The prevalence of MetS was higher in patients with PA than EH in several clinical studies (9, 12) but not in others (10, 11). The controversy appeared to be settled by a recent large meta-analysis including both prospective and retrospective observational, mostly non-matched studies, which found a slightly increased prevalence of diabetes mellitus (DM) and MetS in patients with PA (5); however, the heterogeneity in the diagnosis of MetS, different matching methods, potential selection biases, and the relatively few studies from Asia might affect the results. Therefore, whether MetS prevalence in PA is greater and confers an increased risk of TOD deserves more clinical and research attention, especially in Chinese population.

Notably, there were several important flaws existing in these previous studies, including obvious baseline differences (9, 12), lack of confirmatory tests in diagnosing PA (10). Therefore, in this retrospective study, we tried and alleviated such biases and compared the metabolic profiles between Chinese patients with PA and 1:1 EH control patients individually matched for sex, age, and office systolic BP (SBP) and duration of hypertension. We further investigated the associations between MetS and its individual components and TOD in PA patients to determine whether metabolic disturbances *per se* played any role in causing TOD in the PA patients.

## Materials and Methods

### Study Design and Population

This single-center, case-controlled study was conducted in Sun Yat-sen Memorial Hospital of Sun Yat-sen University (Guangzhou, China). From January 2010 to June 2015, 998 patients who were admitted to the institute for screening of hypertension were enrolled in this study. Written informed consent was obtained from each subject before enrollment. The main reasons for these patients' referral were onset of hypertension at a young age and/or hypertension resistant to conventional antihypertensive therapy, and the purpose was to be screened for a secondary cause of hypertension after comprehensive evaluation. All patients were required to discontinue their antihypertensive drugs, including β-blockers, angiotensin-converting enzyme inhibitors, and angiotensin receptor blockers for ≥2 weeks. Spironolactone was discontinued for ≥6 weeks, and other diuretic drugs were discontinued for ≥4 weeks in order to make aldosterone-to-renin ratio (ARR) test informative. Only α_1_-receptor blockers and non-dihydropyridine Ca^2+^ antagonists (verapamil or diltiazem) were permitted to control BP if necessary, for these agents are known to have a neutral effect on renin and aldosterone levels and not to impair glucose and lipid profiles. Hypokalemia, which reduces aldosterone secretion, should be corrected with oral K^+^ supplements before evaluation. In patients taking oral antidiabetic drugs or lipid-lowering drugs, treatment was withdrawn at least 3 days or 4 weeks before biochemical evaluation if possible, respectively. All patients who recently receive insulin or steroid treatments or with missing information of either matching factors or MetS parameters were excluded from this study. This study protocol was approved by the Ethics Committee of Sun Yat-sen Memorial Hospital.

### Diagnostic Criteria of PA and EH

Our methods for screening and criteria for diagnosing PA and EH were in accordance with the institutional guidelines (13). After withdrawal of medication influencing the renin–angiotensin–aldosterone system and prescription of potassium supplementation, the screening test for PA was positive if the ARR was >25 ng/dl per ng/ml per h in the standing position (13). Then, the diagnosis was confirmed by the failure of aldosterone suppression after captopril challenge test (CCT) and/or saline infusion test (SIT). Based on the safety and convenience of diagnostic tests, we selected the CCT as the first confirmatory test for the patients who screened positive. For CCT, 2 h sitting or standing after taking 25 mg captopril orally, a combination of post-medication plasma aldosterone concentration (PAC) >10 ng/dl with a PRA that remained suppressed or ARR >8.1 ng/dl per ng/ml per h reached a diagnosis of PA, whereas if post-medication PRA elevated >1 ng/ml/h, together with suppression of PAC, the diagnosis of PA was excluded. For patients with inconclusive result after CCT, a second confirmatory test, SIT was performed, and the diagnosis of PA was established if post-SIT PAC >10 ng/dl. Notably, if a patient with spontaneous hypokalemia had undetectable plasma renin with PAC >20 ng/dl, this was most likely PA and confirmatory testing was not necessary. If the patient agreed with the doctor's proposal of surgery, she/he was referred for an adrenal venous sampling (AVS) procedure to make the distinction between unilateral and bilateral adrenal disease. If AVS failed, adrenal high-resolution computerized tomography (CT) with systemic use of a contrast medium and/or magnetic resonance imaging helped determine PA subtypes, which were confirmed by pathology. If no adenoma was indicated pathologically, patients were classified as having PA of undetermined subtype. Specifically, APA was diagnosed according to findings of AVS and/or a combination of CT finding of unilateral hypodense nodules and pathologically confirmed adrenocortical adenoma after laparoscopic adrenalectomy.

Patients in the EH control group were patients with EH referred to our center during the same period. They were considered suitable for analysis when meeting the following criteria: a known history of hypertension with antihypertensive drugs treatment and/or three documented office SBP ≥140 mmHg and/or diastolic BP (DBP) ≥90 mmHg at different days. Secondary causes of hypertension other than PA, such as renovascular hypertension, pheochromocytoma, or Cushing syndrome, were excluded by reviewing records for medical history, physical examination, and a set of laboratory biochemical tests, mainly including ARR, serum and urine cortisol concentration, 1 mg dexamethasone overnight suppression test, plasma-free metanephrines measurement, and/or renal CT angiography as clinically indicated. All the participants were individually matched as pairs between the PA and EH groups according to sex, age (±3 years), and office SBP (±10 mmHg) and duration of hypertension (±2 years). As waist circumference (WC) is part of the MetS definition, body mass index was not considered a matching factor in this study (11).

Resistant hypertension was defined as uncontrolled BP despite the use of ≥3 antihypertensive agents of different classes, including a diuretic, usually thiazide or thiazide-like, a long-acting calcium channel blocker, and a blocker of the renin–angiotensin system, either an angiotensin-converting enzyme inhibitor or an angiotensin receptor blocker, at optimal or maximally tolerated doses (14) when evaluating the medication history of the hypertensive patients.

### BP Measurements

Three BP measurements were obtained in the sitting position, with a validated semiautomatic manometer (Omron 705CP, Japan), leaving a 5 min rest period between measurements. Office BP was determined by calculating the mean of three measured values.

### Definition of MetS

BMI was calculated as weight divided by height squared (kg/m^2^). As for the definition of MetS, it is recommended that the increased WC be judged by ethnically appropriate cutoff points (15). We utilized the definition of MetS endorsed by the Joint Committee for Developing Chinese Guidelines on Prevention and Treatment of Dyslipidemia in Adults (16), which requires at least three of the following findings: (1) abdominal obesity (WC >90 cm in men and >85 cm in women); (2) serum TG ≥1.7 mmol/L (150 mg/dl); (3) HDL-C <1.04 mmol/L (40 mg/dl); (4) SBP ≥130 mmHg or DBP ≥85 mmHg; and (5) impaired fasting glucose (IFG) [fasting plasma glucose (FPG) ≥6.1 mmol/L and <7.0 mmol/L] or impaired glucose tolerance (IGT) [oral glucose tolerance test (OGTT) 2 h plasma glucose (2 h PG) ≥7.8 and <11.1 mmol/L] or DM, a known history of DM, current intake of antidiabetic therapy, and/or two documented FPG ≥7.0 mmol/L or OGGT 2 h blood glucose ≥11.1 mmol/L.

### Biochemical Measurements

FPG, fasting plasma insulin (FINS), OGTT, and glycated hemoglobin (HbA1c) levels were collected to determine glucose homeostasis status. After an overnight 10 h fasting, participants underwent an OGTT. Blood samples were drawn using a polyethylene cannula in an antecubital vein at 0, 30, 60, and 120 min. FPG and FINS indicated the levels of plasma glucose and insulin at 0 min, respectively. Directly after time 0 min, a standard 75 g glucose solution was consumed within 3–5 min. Glucose and insulin samples at each time post-OGTT were collected for analysis. Four surrogate indexes for insulin sensitivity or sensitivity and the islet β-cell function, namely, homeostasis model assessment (HOMA) indexes, the products of FPG and FINS, and two OGTT-derived indexes, were used to evaluate glucose metabolism. HOMA for insulin resistance (HOMA-IR) and insulin sensitivity index (ISI) were calculated to reflect insulin resistance in the body. The HOMA-IR was calculated by the following formula: FPG × FINS/22.5 (17, 18); composite ISI was calculated using the equation: 10,000/$\sqrt{FPG\times FINS\times G\times I}$. *G* and *I* were the means of glucose (mmol/L) and insulin (μU/ml) plasma concentrations during the OGTT 30, 60, and 120 min, respectively (15). A combination of basic insulin secretion index (HOMA-β) and modified β-cell function index (MBCI) were used to assess islet β-cell function. HOMA-β was measured by the equation, 20 × FINS/(FPG – 3.5) (17, 18), and MBCI was calculated using the formula (FPG × FINS)/(G_60_ + G_120_ – 7.0), where G_60_ and G_120_ were the plasma glucose levels (mmol/L) during the OGTT 60 and 120 min, respectively (19).

Serum levels of total cholesterol (TC), TG, HDL-C, and low-density lipoprotein cholesterol (LDL-C) were measured to determine lipid metabolism disorders. Other parameters including serum potassium, sodium, creatinine, 24 h urine sodium, potassium, urinary albumin excretion rate (UAER), and urine specific gravity were also collected for analysis. All the serum and urine samples were measured by a standardized and certified program using an automatic biochemical analyzer (AU5800, Beckman Coulter, USA) at Sun Yat-sen Memorial Hospital.

### Evaluation of TOD Indices

Renal functions were evaluated by estimated glomerular filtration (eGFR) using the CKD-EPI equation, and renal dysfunction was defined as eGFR <60 ml/min per 1.73 m^2^ (20). Microalbuminuria was defined as a UAER ranging from 20 to 200 μg/min. Two-dimensional echocardiography (Acuson Sequoia 512, Siemens Medical Solutions, USA) was performed for the evaluation of LVH, a marker of cardiac damage. The left ventricular mass was calculated by the Devereux equation using parameters from M-mode recordings and normalized by body surface area (g/m^2^), termed as left ventricular mass index (LVMI) (21). Echocardiographic LVH is defined as LVMI ≥115 g/m^2^ for men and ≥95 g/m^2^ for women (22).

### Hormonal Assessment

Blood samples were drawn between 08:00 and 10:00 a.m. for analysis of PAC and PRA after at least 2 h upright posture. PAC, 24 h urine aldosterone, and PRA were assessed by a radioimmunoassay kit using commercial kits (DSL, Webster, TX, USA and DiaSorin, Stillwater, MN, USA, respectively). The intra- and interassay coefficients of variation for PAC at a 11.28 ng/dl concentration were 4.5 and 9.8%, respectively; the normal range was 3.81–31.33 ng/dl. The intra- and interassay coefficients of variation for PRA at 1.6 ng/ml/h were 5.6 and 10%, respectively, and the normal range was 2.63 ± 1.32 ng/ml/h.

### Statistical Analysis

Continuous variables were reported as mean values with standard deviation for normal distribution, median, and interquartile ranges for a skewed distribution. Categorical variables were presented as frequencies or percentages. Student's *t*, Mann–Whitney *U*, or Pearson chi-square test were chosen when appropriate to test for the group differences between PA and EH or APA and IHA or PA with TOD and without TOD groups. Univariate and multivariate logistic regression analyses were used to investigate the association between MetS and TOD in PA. PAC was introduced into the regression model as a normally distributed value, LnPAC. Data were expressed as the odds ratio (OR) and 95% confidence interval (CI). All statistical analyses were performed using SPSS 20 (SPSS Inc., Chicago, IL, USA), and two-tailed *P* < 0.05 were considered statistically significant.

## Results

**Figure 1:**
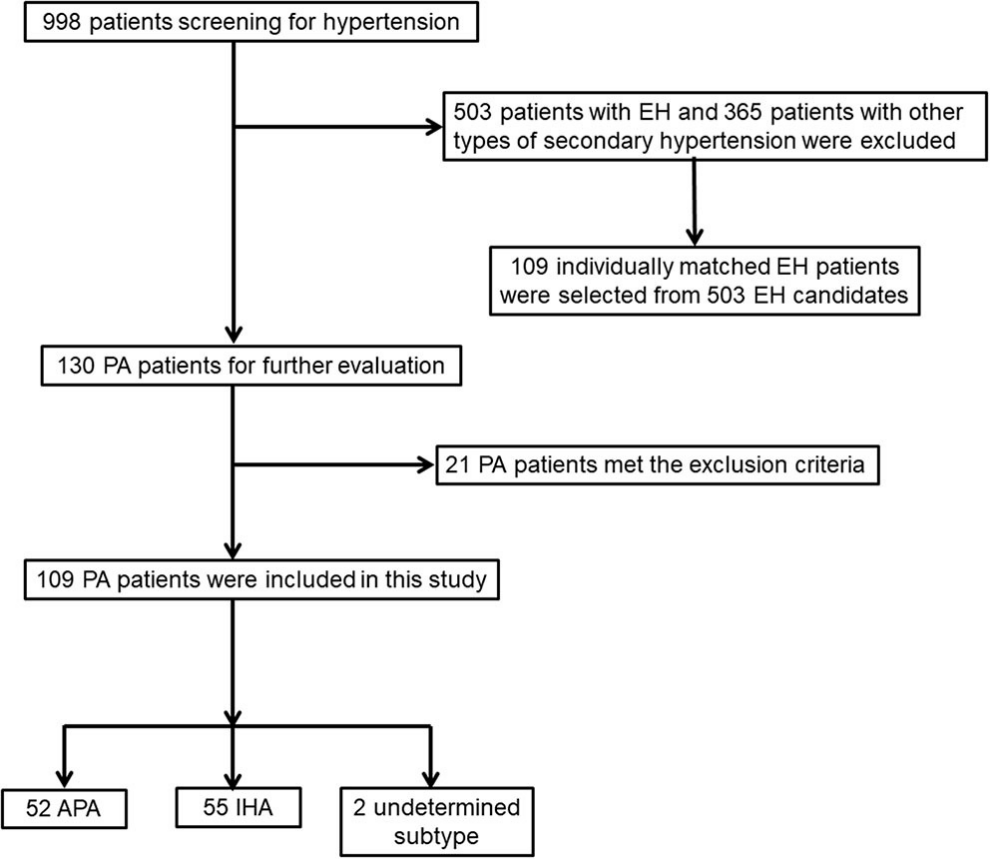
Flowchart of screening and selection process of patients with primary aldosteronism (PA) and essential hypertension (EH).

A total of 998 patients with hypertension were recruited. Of these patients, 130 patients were found to have PA, thus leading to a prevalence of PA in this cohort of 13.0%. Among the 130 PA patients, 21 PA patients were excluded for meeting exclusion criteria; a total of 109 PA cases (mean age, 44.9 ± 9.5 years; 40.4% men) and 109 matched EH controls (mean age, 45.1 ± 9.7 years; 40.4% men) were enrolled in this study (Figure 1). Of the 109 finally included PA cases, 8 patients did not need confirmatory testing (in the setting of spontaneous hypokalemia, undetectable PRA along with PAC >20 ng/dl), 70 were confirmed with CCT, 11 with SIT, and 20 with both tests. APA was diagnosed in 52 patients, and unilateral laparoscopic adrenalectomy was administered: 4 were diagnosed by using AVS, and 48 had unilateral hypodense image in adrenal imaging, which were later confirmed as adrenocortical adenoma by the histological examinations; 33 APA patients achieved a post-operative biochemical cure. IHA was diagnosed in 55 patients, and the remaining 2 patients were of undetermined subtype. As matched beforehand, there were no significant differences for age, sex, SBP, and duration of hypertension between PA and EH patients.

### Comparisons of Characteristics Between Patients With PA and With EH

The demographic, biomedical, and echocardiographic characteristics of patients with PA and EH patients are shown in Table 1. As expected, PA patients had significantly higher levels of serum sodium, PAC, 24 h urine potassium and aldosterone concentrations, and lower serum potassium and PRA than EH controls (*P* < 0.001). BMI and WC were significantly higher in EH than in PA patients (25.8 ± 3.7 vs. 22.4 ± 3.1 kg/m^2^; 88.2 ± 10.6 vs. 80.7 ± 8.3 cm; both *P* < 0.001). Serum levels of TG, LDL-C, 2 h PG, and FINS were significantly lower in PA than in EH patients [1.37 (0.87, 1.78) vs. 1.60 (1.23, 2.47) mmol/L; 2.87 ± 0.75 vs. 3.09 ± 0.78 mmol/L; 7.63 ± 2.60 vs. 8.86 ± 3.34 mmol/L; 7.86 ± 3.62 vs. 9.98 ± 4.54 μU/ml, all *P* < 0.05]. Moreover, compared to EH patients, PA patients had a higher composite ISI [130.21 (67.22, 212.55) vs. 68.12 (43.21, 122.67), *P* < 0.001] but a lower HOMA-IR [1.11 (0.44, 2.00) vs. 2.10 (0.94, 3.22), *P* < 0.001], HOMA-β [79.40 (51.96, 125.84) vs. 139.93 (70.30, 212.45), *P* < 0.001], and MBCI [2.78 (1.43, 4.22) vs. 3.55 (1.67, 6.73), *P* = 0.006]. However, no significant differences were found between PA and EH patients for serum TC, HDL-C, FPG, HbA1c, 24 h urine sodium, the prevalence of resistant hypertension, and history of antihypertensive drugs previously used.

**Table 1:** Comparison of clinical characteristics, metabolic profiles, TOD, and potentially influential medications between PA and EH.

**Characteristics**	**PA (*n* = 109)**	**EH (*n* = 109)**	***P***
**Demographic characteristics**			
Male (*n*, %)	44 (40.4%)	44 (40.4%)	1.000
Age (years)	44.9 ± 9.5	45.1 ± 9.7	0.225
BMI (kg/m^2^)	22.4 ± 3.1	25.8 ± 3.7	<0.001
WC (cm)	80.7 ± 8.3	88.2 ± 10.6	<0.001
Duration of hypertension (years)	4.0 (1.3, 8.5)	4.00 (2.0, 10.0)	0.647
SBP (mmHg)	157.0 ± 16.8	156.2 ± 17.3	0.346
DBP (mmHg)	95.6 ± 11.6	93.3 ± 13.8	0.127
Resistant hypertension (*n*, %)	35 (32.1%)	29 (26.6%)	0.372
**Serum biochemical characteristics**			
Serum potassium (mmol/L)	3.18 ± 0.46	3.86 ± 0.32	<0.001
Serum sodium (mmol/L)	141.97 ± 2.60	140.60 ± 2.99	<0.001
PAC (ng/L)	423.00 (236.20, 701.55)	114.30 (66.75, 168.65)	<0.001
PRA (ng/ml/h)	0.10 (0.03, 0.26)	1.76 (1.00, 2.97)	<0.001
ARR (ng/dl per ng/ml per h)	343.75 (98.05, 2224.17)	7.57 (3.53, 18.55)	<0.001
Creatinine (μmol/L)	95.47 ± 25.54	89.15 ± 19.78	0.020
TG (mmol/L)	1.37 (0.87, 1.78)	1.60 (1.23, 2.47)	<0.001
HDL-C (mmol/L)	1.30 ± 0.32	1.23 ± 0.30	0.080
LDL-C (mmol/L)	2.87 ± 0.75	3.09 ± 0.78	0.022
TC (mmol/L)	4.76 ± 0.94	4.96 ± 1.02	0.084
FPG (mmol/L)	5.28 ± 1.26	5.57 ± 1.33	0.075
2 h PG (mmol/L)	7.63 ± 2.60	8.86 ± 3.34	0.004
FINS (μU/ml)	7.86 ± 3.62	9.98 ± 4.54	<0.001
HbA1c (%)	5.88 ± 1.31	5.95 ± 0.94	0.646
HOMA-β	79.40 (51.96, 125.84)	139.93 (70.30, 212.45)	<0.001
MBCI	2.78 (1.43, 4.22)	3.55 (1.67, 6.73)	0.006
HOMA-IR	1.11 (0.44, 2.00)	2.10 (0.94, 3.22)	<0.001
Composite ISI	130.21 (67.22, 212.55)	68.12 (43.21, 122.67)	<0.001
**Urinary biochemical characteristics**			
24 h urine sodium (mmol/24 h)	125.40 ± 45.04	120.28 ± 45.60	0.312
24 h urine potassium (mmol/24 h)	58.29 ± 25.14	34.22 ± 8.85	<0.001
24 h urine aldosterone (μg/24 h)	29.00 (14.38, 39.31)	7.10 (4.89, 15.05)	<0.001
UAER (μg/min)	19.30 (10.55, 35.70)	9.00 (5.10, 18.20)	<0.001
Microalbuminuria (*n*, %)	51 (46.8%)	21 (19.2%)	<0.001
eGFR (ml/min per 1.73m^2^)	74.92 ± 19.22	80.21 ± 18.46	0.029
Urine specific gravity	1.015 ± 0.005	1.020 ± 0.011	<0.001
**Echocardiographic characteristics**			
LVMI (g/m^2^)	102.36 ± 30.37	92.07 ± 22.93	0.006
LVH (*n*, %)	42 (38.5%)	19 (17.4%)	0.001
**Medication**			
Beta-blockers (*n*, %)	19 (17.4%)	17 (15.6%)	0.715
Diuretics (*n*, %)	27 (24.8%)	26 (23.9%)	0.875

*ARR, aldosterone-to-renin ratio; BMI, body mass index; DBP, diastolic blood pressure; eGFR, estimated glomerular filtration rate; EH, essential hypertension; FINS, fasting serum insulin; FPG, fasting plasma glucose; HDL-C, high density lipoprotein cholesterol; HbA1c, hemoglobin A1c; HOMA-β, basic insulin secretion index; HOMA-IR, homeostasis model assessment for insulin resistance; MBCI, modified β-cell function index; ISI, insulin sensitivity index; LDL-C, low density lipoprotein cholesterol; LVH, left ventricular hypertrophy; LVMI, left ventricular mass index; PA, primary aldosteronism; PAC, plasma aldosterone concentration; 2 h PG, oral glucose tolerance test 2 h plasma glucose; PRA, plasma renin activity; UAER, urinary albumin excretion rate; WC, waist circumference; SBP, systolic blood pressure; TC, total cholesterol; TG, triglycerides; TOD, target-organ damage*.

With regard to TOD, PA patients had significantly higher serum creatinine, UAER levels, and microalbuminuria prevalence than EH controls [95.47 ± 25.54 vs. 89.15 ± 19.78 μmol/L; 19.30 (10.55, 35.70) vs. 9.00 (5.10, 18.20) μg/min; 51 (46.8%) vs. 21 (19.2%), all *P* < 0.05] as well as lower levels of eGFR (74.92 ± 19.22 vs. 80.21 ± 18.46 ml/min per 1.73 m^2^, *P* = 0.029) and urine specific gravity (1.015 ± 0.005 vs. 1.020 ± 0.011, *P* < 0.001). In addition, LVMI and LVH prevalence were significantly higher in PA than in EH patients [102.36 ± 30.37 vs. 92.07 ± 22.93 g/m^2^, 42 (38.5%) vs. 19 (17.4%), both *P* < 0.05].

### Lower Prevalence of MetS and Its Individual Components in Patients With PA Than in EH

**Figure 2:**
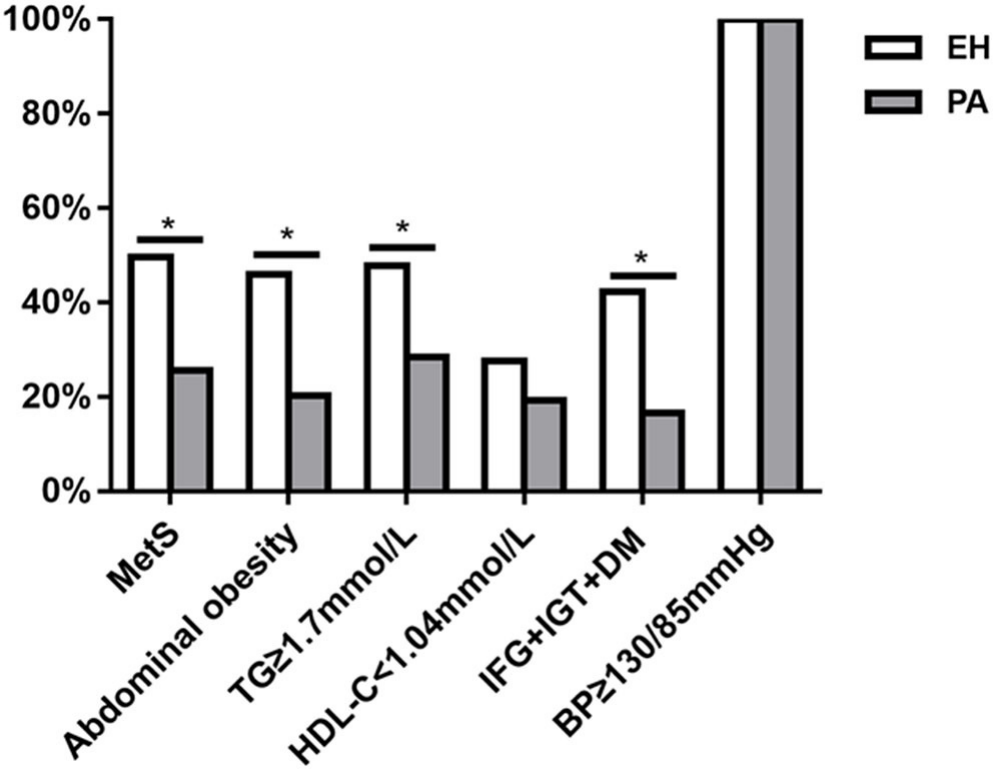
Comparison of the prevalence of metabolic syndrome (MetS) and its individual components between patients with primary aldosteronism (PA) and essential hypertension (EH). *P < 0.05.

As shown in Figure 2, the prevalence of MetS in PA patients was significantly lower than in EH controls [28 (25.6%) vs. 54 (49.5%), *P* < 0.001]. In the PA group, there were 16 cases of MetS diagnosed with any 3 components, 11 with any 4 components, and 1 with 5 components; whereas in EH group, there were 23 cases of MetS diagnosed with any 3 components, 24 with any 4 components, and 7 with 5 components. We thus further compared the distribution of MetS individual components between the two groups (Figure 2). Except hypertension and low HDL-C [109 (100%) vs. 109 (100%); 21 (19.3%) vs. 30 (27.5%), both *P* > 0.05], the prevalence of abdominal obesity, hypertriglyceridemia, and impaired glucose metabolism (IFG + IGT + DM) was significantly lower in PA than those in EH patients [22 (20.2%) vs. 50 (45.9%); 31 (28.4%) vs. 52 (47.7%); 18 (16.5%) vs. 46 (42.2%), all *P* < 0.001). We also investigated the distribution of MetS and its individual components between APA and IHA. As shown in Table 2, the prevalence of global MetS was similar in APA and IHA patients [10 (19.2%) vs. 18 (32.7%), *P* = 0.112], but the prevalence of impaired glucose metabolism (IFG + IGT + DM) and low HDL-C was significantly higher in IHA than in APA patients [4 (7.7%) vs. 14 (25.5%); 5 (9.6%) vs. 16 (29.1%), both *P* < 0.05].

**Table 2:** Comparison of MetS and its individual components between two main PA subtypes.

**Variables**	**APA (*n* = 52)**	**IHA (*n* = 55)**	***P***
MetS (*n*, %)	10 (19.2%)	18 (32.7%)	0.112
Abdominal obesity (*n*, %)	10 (19.2%)	12 (21.8%)	0.741
IFG + IGT + DM (*n*, %)	4 (7.7%)	14 (25.5%)	0.014
Low HDL-C (*n*, %)	5 (9.6%)	16 (29.1%)	0.011
High TG (*n*, %)	12 (23.1%)	19 (34.5%)	0.191

*APA, aldosterone-producing adenoma; DM, diabetes mellitus; HDL-C, high density lipoprotein cholesterol; IFG, impaired fasting glucose; IGT, impaired glucose tolerance; IHA, idiopathic hyperaldosteronism; LDL-C, low density lipoprotein cholesterol; MetS, metabolic syndrome; PA, primary aldosteronism; TG, triglycerides; TOD, target-organ damage*.

### Associations Between MetS and TOD in Patients With PA

We then investigated whether there was a link between MetS and TOD in patients with PA. We divided the PA patients into those with TOD and those without. As shown in Table 3, PA patients with TOD were more often female [24 (66.7%) vs. 20 (27.4%), *P* < 0.001], older (46.9 ± 9.4 vs. 40.8 ± 8.3 years, *P* = 0.021), with a longer duration of hypertension [6.0 (2.0, 10.0) vs. 1.9 (1.0, 4.0) years, *P* < 0.001] and higher LnPAC (6.14 ± 0.76 vs. 5.62 ± 0.89 ng/L, *P* = 0.002) than those without TOD. As expected, compared to PA patients without TOD, those with TOD had significantly lower eGFR and urine specific gravity and higher LVMI and UAER (all *P* < 0.05). However, MetS and its individual components, as well as PA subtype distribution, were not significantly different in PA patients with and without TOD (all *P* > 0.05). Univariate logistic regression analysis showed that age, sex, duration of hypertension, and LnPAC were significantly associated with TOD in patients with PA (Table 4); female gender (OR, 0.077; 95% CI, 0.022–0.256; *P* < 0.001) and LnPAC (OR, 2.257; 95% CI, 1.068–4.767; *P* = 0.033) remained significantly associated with TOD on multivariate logistic analysis (Table 4).

**Table 3:** Comparison of characteristics between PA with TOD and without TOD.

**Variables**	**PA with TOD (*n* = 73)**	**PA without TOD (*n* = 36)**	***P***
Male (*n*, %)	20 (27.4%)	24 (66.7%)	<0.001
Age (years)	46.9 ± 9.4	40.8 ± 8.3	0.021
Duration of hypertension (years)	6.0 (2.0, 10.0)	1.9 (1.0, 4.0)	<0.001
TG (mmol/L)	1.37 (0.86, 2.08)	1.38 (0.93, 1.72)	0.869
HDL-C (mmol/L)	1.28 ± 0.29	1.34 ± 0.37	0.327
FPG (mmol/L)	5.24 ± 1.04	5.35 ± 1.63	0.678
HOMA-β	77.73 (49.25, 77.73)	83.23 (58.74, 161.50)	0.584
MBCI	2.67 (1.38, 3.94)	3.44 (2.11, 4.90)	0.125
HOMA-IR	1.11 (0.45, 1.99)	1.13 (0.41, 2.09)	0.782
Composite ISI	123.50 (61.30, 213.05)	135.41 (102.06, 211.89)	0.190
UAER (μg/min)	27.30 (18.60, 43.30)	9.95 (3.38, 18.53)	<0.001
Urine specific gravity	1.013 ± 0.005	1.017 ± 0.006	<0.001
MetS (*n*, %)	18 (24.7%)	10 (27.8%)	0.726
Abdominal obesity (*n*, %)	12 (16.4%)	10 (27.8%)	0.165
IFG + IGT + DM (*n*, %)	13 (17.8%)	5 (13.9%)	0.604
Abnormal lipid profiles (*n*, %)	28 (38.4%)	12 (33.3%)	0.609
eGFR (ml/min per 1.73 m^2^)	71.64 ± 18.62	81.56 ± 18.93	0.011
LVMI (g/m^2^)	108.36 ± 34.39	90.19 ± 13.60	<0.001
LnPAC (ng/L)	6.14 ± 0.76	5.62 ± 0.89	0.002
PA subtypes			0.407
APA (*n*, %)	37 (50.6%)	15 (41.7%)	
Bilateral PA (*n*, %)	35 (47.9%)	20 (55.6%)	

*TOD was defined as eGFR <60 ml/min per 1.73 m^2^, microalbuminuria, and/or LVMI >115 g/m^2^ (male) or >95 g/m^2^ (female). Abdominal obesity was defined as WC >90 cm (male) or >85 cm (female). Abnormal lipid profiles were defined as serum TG ≥1.7 mmol/L (150 mg/dl) and/or HDL-C <1.04 mmol/L (40 mg/dl). APA, aldosterone-producing adenoma; eGFR, estimated glomerular filtration rate; DM, diabetes mellitus; FPG, fasting plasma glucose; HDL-C, high density lipoprotein cholesterol; IFG, impaired fasting glucose; IGT, impaired glucose tolerance; HOMA-β, basic insulin secretion index; HOMA-IR, homeostasis model assessment for insulin resistance; ISI, insulin sensitivity index; LDL-C, low density lipoprotein cholesterol; LVMI, left ventricular mass index; MBCI, modified β-cell function index; MetS, metabolic syndrome; PA, primary aldosteronism; PAC, plasma aldosterone concentration; UAER, urinary albumin excretion rate; TG, triglycerides; TOD, target-organ damage*.

**Table 4:** Associations between potential risk factors and TOD in patients with PA.

**Variables**	**Univariate logistic regression analysis**		**Multivariate logistic regression analysis**	
	**OR (95% CI)**	***P***	**OR (95% CI)^**#**^**	***P***
Age	1.082 (1.029, 1.138)	0.002	1.083 (0.995, 1.178)	0.064
Sex (male = 1, female = 0)	0.189 (0.080, 0.447)	<0.001	0.077 (0.022, 0.256)	<0.001
Duration of hypertension	1.284 (1.117, 1.477)	<0.001	1.210 (0.993, 1.475)	0.059
MetS	0.851 (0.345, 2.099)	0.726	–	–
Abdominal obesity	0.511 (0.197, 1.331)	0.170	–	–
IFG + IGT + DM	1.343 (0.439, 4.112)	0.605	–	–
Abnormal lipid profiles	1.244 (0.538, 2.878)	0.609	–	–
LnPAC	2.167 (1.284, 3.658)	0.004	2.257 (1.068, 4.767)	0.033

*TOD was defined as estimated glomerular filtration rate <60 ml/min per 1.73 m^2^ microalbuminuria and/or left ventricular mass index >115 g/m^2^ (male) or >95 g/m^2^ (female). Abdominal obesity was defined as waist circumference >90 cm (male) or >85 cm (female). Abnormal lipid profiles were defined as serum triglycerides ≥1.7 mmol/L (150 mg/dl) and/or high-density lipoprotein cholesterol <1.04 mmol/L (40 mg/dL). CI, confidence interval; DM, diabetes mellitus; IFG, impaired fasting glucose; IGT, impaired glucose tolerance; MetS, metabolic syndrome; OR, odds ration; PA, primary aldosteronism; PAC, plasma aldosterone concentration; TOD, target-organ damage*.

## Discussion

The matched case–control study shows that Chinese patients with PA exhibited more severe TOD but have a lower prevalence of MetS than control patients with EH. Rather than MetS and its individual components, elevated plasma aldosterone and female gender were significantly associated with TOD in PA patients.

Consistent with previous studies (4, 23), our study showed that compared to matched EH controls, PA patients exhibited more renal damage indicated by reduced eGFR and greater prevalence of microalbuminuria, as well as more cardiac damage indicated by an increased prevalence of LVH. On the one hand, there is accumulating evidence including our previous researches, which support that many detrimental effects of aldosterone excess are involved in causing pre-clinical TOD in the PA patients; they include inflammation, oxidative stress, endothelial dysfunctions, and vascular calcification (7, 24–27). On the other hand, a majority of patients with MetS, a cluster of risk factors, tend to develop structural and functional abnormalities of target organs, which precede cardiovascular complications (28, 29). However, the relationship between PA and MetS remains controversial and plausible (30).

It is worth noting that increased prevalence of impaired glucose metabolism and MetS in patients with PA has been mostly described in population-based studies, which used the general population as control rather than patients with EH (12, 31). Studies comparing abnormalities in glucose metabolism and MetS in patients with PA and those with EH have yielded conflicting results (9–12). These studies were mostly performed on Western populations, and there is still a lack of data from Asian populations, especially from China (5). We adopted the definition of MetS parameters based on the epidemiology of metabolic profiles of Chinese population (16), which are different from those measured in Caucasians (12, 32), including a smaller cutoff of WC and detailed definitions for abnormal glucose metabolism. Such definitions were of course the same for PA patients and their matched EH controls. The final results may thus be easily compared with those found in previous studies (9, 10, 12). Our study convincingly showed that Chinese individuals with PA had lower prevalence of MetS and its individual components than those with EH. These results may appear contrary to the findings of the recent meta-analysis, indicating that MetS was significantly more frequent in PA patients than in the control population (5). However, there were only two matched studies included for meta-analysis that had compared the prevalence of MetS in patients with PA and EH. One small-sized study showed that there was no significant difference in the prevalence between patients with PA and matched EH controls (*P* = 0.138) (11). Another study from German Conn's Registry (12) included in the meta-analysis claimed individually 183 PA and 183 EH patients matched on three matching factors: sex, age, and BP; however, the baseline data showed a significantly higher SBP in patients with PA than in EH (153.3 ± 20.0 vs. 148.5 ± 20.0 mmHg; *P* = 0.03). In addition, there was no information with regard to the duration of hypertension in patients with PA and EH. Therefore, potential baseline confounders might have contributed to greater frequency of MetS in PA and could not be excluded (12). PA subtypes may also play a role in the MetS distribution: patients with APA (but not IHA) had a lower prevalence of MetS than those with EH (33). In our study, nearly half of the PA patients had APA; similarly, the prevalence of some individual components of MetS, in particular parameters of impaired glucose metabolism, were significantly lower in APA than in IHA patients. Such differences may be due to distinct pathophysiology of the different PA subtypes (34).

A recent multi-institutional cohort study in Japan investigated the prevalence and causes of DM in patients with PA (35). Notably, among this multi-institutional collaboration, the Kyoto Medical Center cohort showed a higher prevalence of DM in PA patients compared to EH controls matched for age and sex. However, the difference became non-significant when comparing PA patients without suspected subclinical hypercortisolism to the matched EH controls; this finding suggests an important role of hypercortisolism for glucose metabolism disorder in PA (35). In our study, we have used four surrogate markers to evaluate insulin resistance or sensitivity: our results indicated that although Chinese PA patients had greater damage in pancreatic β-cells characterized by reduced HOMA-β and MBCI, they exhibited less severe insulin resistance than their matched EH controls. Our results are consistent with a previous small cohort study, which showed that PA patients had higher glucose levels; in that study, hyperglycemia could not be explained by decreased insulin sensitivity because the insulin levels and the HOMA-IR index were similar in both groups, whereas it could be explained by a lower β-cell function due to aldosterone excess, as HOMA-β index was lower in PA patents than in EH patients (17). Our results are also in agreement with previous studies in PA, which showed that an impairment of islet β-cell and first-phase insulin secretion was significantly improved after adrenalectomy (11, 36, 37). Based on these and our findings, we speculate that declined insulin secretion resulting from more pronounced islet β-cell damage, rather than insulin resistance, may play an important role in the progression of impaired glucose metabolism in PA individuals. Conversely, in the EH population, however, we believe that decreased insulin sensitivity or increased insulin resistance may mainly contribute to hyperglycemia and/or DM and to MetS (38).

In our study, neither MetS nor its individual components were risk factors of TOD in PA setting, although PA patients were more prone to TOD than EH patients, which emphasizes the need to treat PA as a unique cardiovascular risk factor, as it can be either surgically cured or treated with a mineralocorticoid receptor antagonist. A recent meta-analysis comparing PA and EH showed that patients with APA may have lower long-term mortality than EH patients due to the better recovery of adrenalectomy (39). Besides well-recognized risk factors like hyperaldosteronism (27), our results also showed that female gender was a potential determinants of subclinical changes. Gender differences of TOD in hypertension have been also described, but contradictory results are available in the literature. A cross-sectional survey enrolling 876 individuals showed that women had greater difference between central and peripheral SBP than men. Thus, given similar peripheral BP, women were possibly at higher risk for developing TOD (40). Data from the Losartan Intervention for Endpoint (LIFE) study demonstrated less pronounced LVH regression in women than in men during antihypertensive therapy, even after adjustments for confounders (41). Further prospective studies are needed to unravel this aspect in PA setting.

Some potential limitations of the present study should be highlighted. It is a hospital-based retrospective study, and selection bias cannot be completely excluded. However, the prevalence of resistant hypertension in PA and EH patients was similar, which made it less likely to be a confounder. It was very recently well-shown that PA could be unrecognized among patients with EH and that confirmatory tests, such as an oral sodium suppression test, should be performed before formally diagnosing EH (42); we must stress that the overwhelming majority of patients included in our study had confirmatory tests to diagnose or exclude PA. There was no control group without hypertension, which did not allow us to compare the prevalence of MetS in PA patients to that in the normal non-hypertensive population; however, this was not the objective of our study. The diagnosis of AVS is the gold standard test used in distinguishing between unilateral and bilateral adrenal disease (13); adrenal imaging is now considered accurate and undoubtedly less invasive when AVS is unavailable (43). Insulin resistance and the β-cell function were assessed and expressed by the HOMA indexes and the OGTT-derived indexes, and whether these surrogate indexes can offer the same information as glucose clamps in the setting of PA needs to be verified.

In conclusion, our study suggests that excessive TOD experienced by Chinese patients with PA compared to those with EH is mainly due to the detrimental effects of hyperaldosteronism, whereas MetS or its individual components might be less influential. Recent findings of the coexistence of glucocorticoid and aldosterone excess in cases of PA (35, 44, 45) highlight the necessity for further studies to assess the presumed link between aldosterone excess and various other metabolic parameters; the significant association we found with female gender could find its origin in such disturbances.

## Data Availability Statement

The raw data supporting the conclusions of this article will be made available by the authors, without undue reservation.

## Ethics Statement

The studies involving human participants were reviewed and approved by the Ethics Committee of Sun Yat-sen Memorial Hospital. Written informed consent to participate in this study was provided by the participants prior to enrollment.

## Author Contributions

S-LZ and P-ML conceived and designed the study. YG, Q-LF, J-YT, and LY collected and managed the data. S-LZ and J-WG analyzed the data and wrote the manuscript. P-ML, J-FW, and HC reviewed and edited the manuscript. All authors read and approved the manuscript.

### Conflict of Interest

The authors declare that the research was conducted in the absence of any commercial or financial relationships that could be construed as a potential conflict of interest.
